# The impact of interpersonal reporting heterogeneity on cross-country differences in Healthy Life Years in Europe

**DOI:** 10.1093/eurpub/ckad142

**Published:** 2023-08-22

**Authors:** Marc Luy, Paola Di Giulio, Yuka Minagawa

**Affiliations:** Vienna Institute of Demography (OeAW), Wittgenstein Centre for Demography and Global Human Capital (IIASA, OeAW, University of Vienna), Vienna, Austria; Vienna Institute of Demography (OeAW), Wittgenstein Centre for Demography and Global Human Capital (IIASA, OeAW, University of Vienna), Vienna, Austria; Sophia University, Tokyo, Japan

## Abstract

**Background:**

The European Union has used Healthy Life Years (HLY) as an indicator to monitor the health of its aging populations. Scholarly and popular interest in HLY across countries has grown, particularly regarding the ranking of countries. It is important to note that HLY is based on self-assessments of activity limitations, raising the possibility that it might be influenced by differences in health reporting behaviours between populations, a phenomenon known as differential item functioning (DIF).

**Methods:**

We estimated DIF-adjusted HLY at age 50 for Belgium, France, Germany, Greece, Italy, the Netherlands, Spain, and Sweden to determine the extent to which differences in HLY might be influenced by reporting heterogeneity across countries. We used anchoring vignettes, taken from the 2004 Survey of Health, Ageing and Retirement in Europe, to estimate DIF-adjusted prevalence rates of activity limitations measured by the Global Activity Limitations Indicator (GALI). The Sullivan method was used to calculate DIF-adjusted HLY.

**Results:**

Changes in HLY before and after adjustment ranged from a 1.20-year decrease for men in Italy to a 1.61-year increase for women in Spain. Adjustment for DIF produced changes in the rankings of the countries by HLY, with upward and downward movements of up to three positions.

**Conclusion:**

Our results show that DIF is likely to affect HLY estimates, thereby posing a challenge to the validity of comparisons of HLY across European countries. The findings suggest that HLY should be used to monitor population health status within a country, rather than to make comparisons across countries.

## Introduction

Health expectancy (HE) has been widely used as a comprehensive measurement of the health status of populations. It is an extension of traditional life expectancy and combines information on mortality and morbidity.^1^ In 2005, the European Union (EU) adopted HE under the name of Healthy Life Years (HLY) as its structural indicator on health, which it defined as the average remaining years of life spent free from activity limitations.^2^^,^^3^ It is used to track the levels and trends of population health and to assess the progress of its public health programmes among the member states. The HLY indicator has an important bearing on EU social policy, such as future medical and care expenditures and pension provision, as it provides information on the physical health of its older populations.^4^ Also, HLY was incorporated into the Europe 2020 strategy, which included the EU Innovation Partnership on Active and Healthy Ageing (EIP-AHA) with the headline target to increase the average healthy lifespan in the EU by two years by 2020.^5^

Analyzing HLY across European countries is important for two key reasons. First, there are different trends in mortality and morbidity across countries as well as across regions,^6^^,^^7^ and second, past evidence has suggested that inequalities in socioeconomic development between EU member states translate into differentials in HLY.^4^^,^^6^^,^^8^ Cross-country variations in HLY have therefore generated a substantial interest among policy makers, public health officials, and journalists. Of particular interest is the ranking of the countries by HLY. In this context, different rankings in HLY tend to be interpreted as differences in population health status. It is important to note, however, that HLY is generated on the basis of the Global Activity Limitation Indicator (GALI), i.e. people’s self-assessments of long-term activity limitations due to health problems.^9^^,^^10^ This point raises a question over the validity of cross-national comparisons of HLY. Prior research has suggested that subjective evaluations of health might be influenced by individual, situational, and cultural factors, including differences in reporting behaviours between populations, a phenomenon known as ‘differential item functioning’ (DIF).^11^

The present study addresses this issue by estimating DIF-adjusted HLY at age 50 for eight European countries and explores how HLY may change after adjusting for DIF. To achieve this goal, we use anchoring vignettes to control for the effect of DIF on HLY. The vignette approach corrects intergroup heterogeneity in reporting behaviours and has been widely used to identify DIF within various topics, such as student performance,^12^ life satisfaction,^13^ job satisfaction,^14^ and economic welfare.^15^ Importantly, health researchers have become increasingly interested in using anchoring vignettes to make more valid comparisons of health across populations^16^^,^^17^ as well as among population subgroups.^18–20^ However, to our knowledge, there have been no studies, prior to this one, that have employed anchoring vignettes in evaluating HE. With this research, we contribute to the existing literature by empirically testing the role of interpersonal reporting heterogeneity in HLY in the European context.

While our methods in this study are innovative, the estimation of DIF-adjusted HLY is based on a number of assumptions, and this poses restrictions on the applicability of our approach. These limitations are, however, unavoidable, as we will explain in greater details in the following section. The current results may only be interpreted as approximate estimates of HLY, and our methods should neither be interpreted as a correction of DIF, nor as a proposal to improve the indicator. The objective of this study is to test whether HLY values are subject to a bias due to DIF and whether this bias can be large enough to affect the ranking of European countries by HLY.

## Methods

The GALI, which is the underlying health indicator of the EU’s HLY indicator, is based on the survey question ‘For at least the past six months, to what extent have you been limited because of a health problem in activities people usually do?’ The possible responses are ‘severely limited’, ‘limited but not severely’, and ‘not limited at all’. For the calculation of HLY, the healthy state is defined exclusively by the response category of ‘not limited at all’, whereas ‘severely limited’ and ‘limited but not severely’ are defined as unhealthy.

To adjust for the effect of a possible bias due to interpersonal reporting heterogeneity across populations, we adjusted GALI with anchoring vignettes. Anchoring vignettes are brief, hypothetical descriptions of fictional characters who manifest certain health problems to a lesser or greater degree.^11^ The Survey of Health, Ageing and Retirement in Europe (SHARE) included 27 health vignettes in the first wave, from 2004, for a sub-sample of 4372 individuals from Belgium (548), France (876), Germany (495), Greece (657), Italy (437), the Netherlands (517), Spain (429), and Sweden (413).^21–23^ The SHARE vignettes cover seven different health traits, with three vignettes each from the traits ‘bodily aches or pains’, ‘difficulty with sleeping’, ‘problem with moving around’, ‘difficulty with concentrating and remembering’, ‘problem because of shortness of breath’, ‘problem with feeling sad, low, or depressed’, and nine vignettes from the trait ‘limitations with the kind or amount of work one could do’. Respondents were asked to evaluate the severity of the described health problems, and response categories included ‘none’, ‘mild’, ‘moderate’, ‘severe’, and ‘extreme’. In addition to the evaluations of the vignette characters’ health conditions, respondents were also asked to rate their own health for the same seven health traits using the same answer scale. Examples for these vignettes and the corresponding questions for the respondents themselves can be found in Supplementary material A.

The adjustment for DIF in HLY would require vignettes designed for the GALI. However, such vignettes are not available. The GALI question is formulated broadly with the aim to capture all kinds of health-related limitations in the daily activities that people usually experience.^9^^,^^10^ In contrast, vignettes can describe only very specific health problems. Nonetheless, taken together, the seven health traits of the vignettes are likely to cover the most important health problems experienced by people who report a limitation in the GALI question. Based on this assumption, we tested which health traits were statistically associated with activity limitations in the GALI. In line with EU’s HLY indicator, we dichotomized the health information from the GALI question into healthy (‘not limited at all’) and unhealthy (‘limited, but not strongly’ and ‘strongly limited’). For consistency, we classified also the health traits into the two categories healthy (‘none’ and ‘mild’) and unhealthy (‘moderate’, ‘severe’, and ‘extreme’). We applied least absolute shrinkage and selection operator (lasso) logistic regression and analyzed data separately by age group (50–59, 60–74, and 75 and above) and gender, including the country variable as a control. All analyses were weighted.

For the estimation of DIF-adjustment factors, we used only those vignettes of the health traits that were selected by the lasso regression to be associated with GALI (see Supplementary material B for more details). From the selected vignettes, we calculated for each country, as well as for all countries combined, the corresponding proportions of vignette characters that the respondents assigned as having health problems. The proportions of the total sample were used as a standard, and the age-, gender- and country-specific adjustment factors *w* were calculated from:


$$w_{\mathrm{ijk}}=Q_{ij}/Q_{\mathrm{ijk}}\;$$

where *Q* is the proportion of respondents stating that the health condition described in the selected group of vignettes is moderate, severe, or extreme, *i* is gender (women and men), *j* is the age group to which the selection of vignettes refers (50–59, 60–74, 75+), and *k* is the country (Belgium, France, Germany, Greece, Italy, the Netherlands, Spain, Sweden). A more detailed description of the calculation of the DIF-adjustment factors is presented in Supplementary material B, and the selected health traits for *Q* can be found in Supplementary material C.

In the next step, we adjusted the age, gender, and country-specific GALI prevalence values for DIF by multiplying the unadjusted GALI prevalence values with the corresponding adjustment factors, using *w_i_*_, 50–59,__*k*_ for the age groups 50–54 and 55–59, *w_i_*_, 60–74,__*k*_ for the age groups 60–64, 65–69 and 70–74, and *w_i_*_, 75+,__*k*_ for the age groups 75–79 and 80+. This procedure increased the GALI prevalence in the case where the respondents in a given country were more optimistic about health problems than those in all countries combined, and it decreased it if they were more pessimistic.

Finally, the Sullivan^24^ method was used to estimate DIF-adjusted and unadjusted HLY for men and women at age 50. We used the adjusted and unadjusted prevalence rates of GALI limitations as input into period life tables. GALI prevalence rates were derived from the 2005 European Union Statistics on Income and Living Conditions (EU-SILC).^25^ We used the 2005 EU-SILC, as it is the first year for which these data are available for the EU member states, including the eight countries of the present study. We chose to use the EU-SILC, as it is used for the estimation of HLY by Eurostat. Life tables were taken from the Human Mortality Database.^26^

## Results

**Figure 1:**
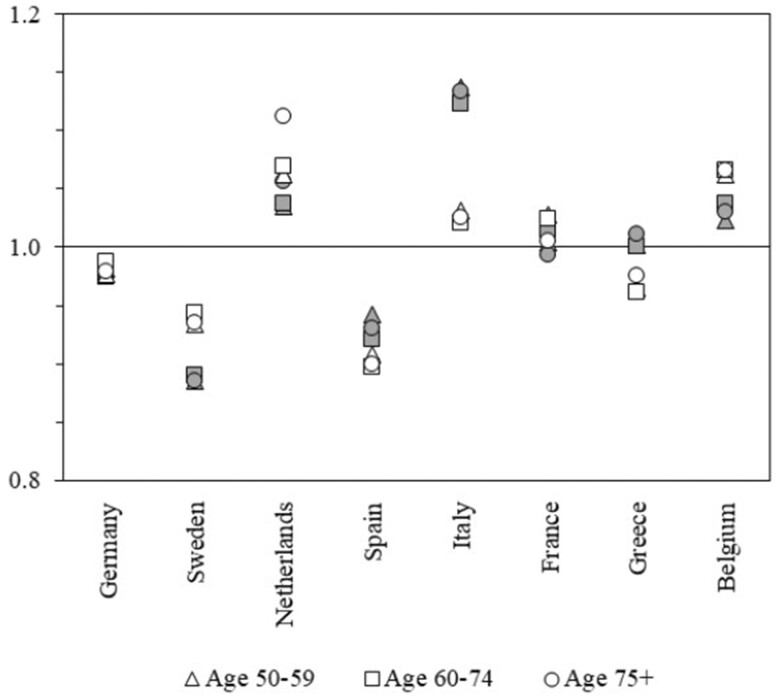
Country-specific adjustment factors for women (white) and men (grey) for the prevalence of self-reported limitations according to the GALI-question, separated by age. Source: Authors’ own calculations with data from SHARE 2004.

Figure 1 presents the estimates of DIF-adjustment factors for the prevalence of GALI limitations, stratified by gender and age group. Values above 1.0 indicate that the respondents from the country assigned a lower number of vignettes to having health problems compared with the total sample of the eight countries, while values below 1.0 suggest a higher number of vignettes. The larger the deviation from 1.0, the larger the deviation in the number of vignettes assigned to health problems. In the entire sample, the adjustment factors varied from 0.89 for men aged between 50 and 59 in Sweden to 1.14 for men of the same age group in Italy. The respondents from Germany and France agreed almost perfectly with the overall sample in their assessments of the vignettes, as seen in the closeness of their adjustment values to 1.0. Perhaps the most startling features of these results are the consistency in reporting behaviours among different demographic groups. Across all countries except Greece, adjustment factors are consistently above or below 1.0 for men and women and across age groups. In Greece, the adjustment factors for men lie minimally above 1.0, whereas those for women are slightly below 1.0.

We used these estimates of adjustment factors to compute gender-specific DIF-adjusted HLY at age 50 (tables 1 and 2). These tables also show the unadjusted HLY values, differences in HLY before and after adjustment for DIF as well as country rankings. We begin our results by focusing on changes in absolute values of HLY before and after adjustment for DIF. Among women, the difference between adjusted and unadjusted HLY ranges from a 1.11-year decrease in the Netherlands to a 1.61-year increase in Spain. Among men, the results vary from a 1.20-year decrease in Italy to a 1.15-year increase in Sweden.

**Table 1:** Estimates of Healthy Life Years (HLY) without and with adjustment for the differential item functioning (DIF) at age 50 for eight European countries, 2005, women

	DIF-unadjusted		DIF-adjusted		Difference adjusted- unadjusted
Country	HLY	Rank	HLY	Rank	
Belgium	19.02	6	18.08	7	−0.94
France	20.24	4	20.01	5	−0.23
Germany	13.89	8	14.25	8	+0.36
Greece	21.35	1	21.74	1	+0.39
Italy	21.28	2	20.95	3	−0.33
Netherlands	20.09	5	18.98	6	−1.11
Spain	18.84	7	20.45	4	+1.61
Sweden	20.37	3	21.21	2	+0.84

Source: Authors’ own calculations based on the data from the SHARE 2004, EU-SILC 2005, and HMD 2005.

**Table 2:** Estimates of Healthy Life Years (HLY) without and with adjustment for the differential item functioning (DIF) at age 50 for eight European countries, 2005, men

	DIF-unadjusted		DIF-adjusted		Difference adjusted- unadjusted
Country	HLY	Rank	HLY	Rank	
Belgium	18.85	6	18.55	6	−0.30
France	18.31	7	18.27	7	−0.04
Germany	13.64	8	13.98	8	+0.34
Greece	20.07	3	20.03	2	−0.04
Italy	20.94	1	19.74	4	−1.20
Netherlands	20.09	2	19.70	5	−0.39
Spain	19.25	5	19.97	3	+0.72
Sweden	19.98	4	21.13	1	+1.15

Source: Authors’ own calculations based on the data from the SHARE 2004, EU-SILC 2005, and HMD 2005.

Changes in HLY lead to remarkable changes in differences in HLY between countries and in country rankings by HLY. Among women, the gap between first and second place widens after adjusting for DIF. While there is a 0.07-year difference in unadjusted HLY between Greece (ranked first) and Italy (ranked second), the gap between the top two countries, namely Greece (ranked first) and Sweden (ranked second), increases to 0.53 years after adjusting for DIF. The largest change in the ranking is observed for Spain, moving up from seventh to fourth place. There are downturns in the rankings for Belgium (from sixth to seventh), France (from fourth to fifth), Italy (from second to third), and the Netherlands (from fifth to sixth). The rankings of Greece (ranked first) and Germany (ranked eighth) remain unchanged. For men, the bottom three countries, including Belgium (ranked sixth), France (ranked seventh), and Germany (ranked eighth), remain unchanged after adjusting for DIF. Although Italy has the highest level of HLY before adjustment, it moves down to fourth place due to statistical adjustment for DIF. Sweden is ranked first in terms of men’s DIF-adjusted HLY, followed by Greece. Spain moves up from fifth to third place. Italy and the Netherlands move down by three places, from first to fourth and second to fifth, respectively.

## Discussion

Heterogeneity in reporting behaviours challenges the validity of comparisons of self-ratings of health between populations and population subgroups, an issue known as DIF.^11^ A failure to account for the impact of DIF, therefore, may lead to errors in the evaluations of population health status. There is strong evidence of inequalities in HLY across European countries,^4^^,^^6^^,^^8^ but the existing country rankings are based on comparisons of absolute values of HLY that do not consider differences in health reporting behaviours among populations. To address this point, the present study used anchoring vignettes as a means to statistically control for the effect of DIF and estimated DIF-adjusted HLY at age 50 for eight selected EU countries. We found notable changes not only in the absolute values in HLY after adjusting for DIF, which ranged from a 1.20-year decrease for men in Italy to a 1.61-year increase for women in Spain, but also in the country rankings. Among women, Greece continued to be ranked first and Germany was ranked bottom even after controlling for DIF. All countries except these two swapped ranking positions, with Spain improving by as many as three places. The DIF-adjustment resulted in changes in the rankings for most countries among men as well. For instance, Italy was replaced by Sweden at the top position, the country with the largest rise in men’s ranking after DIF-adjustment, from fourth to first place.

The present findings offer evidence that both the number of life years spent in good health, i.e. HLY, and the ranking of the countries by HLY may be subject to interpersonal reporting heterogeneity among populations. Past research has suggested how individual, situational, and cultural factors influence the process of self-assessments of health, including individuals’ sociodemographic characteristics, knowledge about specific diseases, the reference group used in health evaluation, and the cultural understanding and expectations of health.^27^^,^^28^ Further, existing evidence has reported how variations in HLY across Europe mirror underlying heterogeneity in the socioeconomic compositions of subpopulations, namely education levels.^8^ While these factors make intergroup comparisons of self-ratings of health difficult, the current results further demonstrated how cultural, social, normative, or linguistic differences in health reporting styles influence HE. It is therefore important to consider DIF as one of the key contributors to variations in HLY across European countries. Making cross-country comparisons of health is indeed challenging in the European context, given the various different languages used among European populations. Our findings have important implications as to how to use HLY in the European context; given a bias introduced by heterogeneity in health reporting behaviours, HLY may better be used to monitor trends and changes in population health or to evaluate health differentials within one country. We therefore urge caution when making comparisons across countries, considering a potential bias from DIF.

The vignettes approach has been adopted in cross-group comparisons of various health measurements,^16^^,^^19^^,^^20^ but this is the first study, to the best of our knowledge, to apply anchoring vignettes to HE. There is a large literature on methods and applications to identify DIF by modelling anchoring vignettes and respondents’ health characteristics.^11^ Our approach is different in that we used the information from the anchoring vignettes to adjust for GALI, which is not covered by the vignettes’ traits and measures health status with a different scale. Although instructive, our results must be interpreted in light of the study’s limitations. The first limitation has to do with the data. We utilized the 2004 SHARE data, because several anchoring vignettes for each health trait are available only in this first wave. The second wave of the SHARE (2006–7) also included questions about vignettes, but those on limitations were restricted to respondents in working age. We therefore relied on wave 1 of the SHARE. Yet, using outdated data does not limit the paper’s main objective to test whether DIF might affect the country ranking in HLY. We anticipate that the substantive conclusions of this paper would be similar even with more recent data, given that the impact of differences in health reporting styles, such as the shared linguistic or cultural understanding of health, is unlikely to change with time.^28^ Also, related to the first point, the second limitation of this study is that only eight countries, the total number of participating countries in the first wave of the SHARE, were included in the analysis. The inclusion of more countries, ideally all EU member states, would offer a more comprehensive picture of differences in health reporting behaviours across Europe and its effects on HLY. Information for Eastern Europe would be particularly valuable, given the existing gap in HLY between Eastern and Western Europe.^2^^,^^4^^,^^6^

The third limitation concerns the discrepancy between vignettes and the underlying health indicator of HLY, i.e. GALI. Given that there are no vignettes directly attached to global activity limitations, we instead used the available health vignettes’ traits to estimate adjustment factors. The vignette health items are likely to cover the key domains of health, but there might be other health conditions linked to activity limitations. Vision problems and hearing impairment, for instance, are critical for performing daily activities, especially those involving interpersonal interactions,^29^^,^^30^ but these items are not available in the vignette questions of the SHARE.

The fourth and perhaps most critical point regarding our analysis is about response categories for the GALI and vignette questions. Following the EU official definition of health, we focused on GALI, and the participants who chose the response category of ‘not limited’ were considered as being healthy. Contrariwise, in the vignettes’ health traits, we used the responses of ‘none’ and ‘mild’ problems to define a healthy state. Ideally, the response category of ‘no health problems’ in the vignettes’ questions would have allowed us to establish a nearly identical definition of health. This was methodologically impossible, however, since all the fictional characters in the vignettes manifest certain health problems, and there are not enough vignettes assessed a state free from any health problems. Using the assessment of ‘mild’ problems to define a healthy state was therefore the only way to construct healthy and unhealthy states for the vignette characters. While this strategy is far away from being perfect, it helped us to address the issue of the discrepancy in response categories between the GALI and vignette questions, making a DIF-adjustment of HLY possible. Moreover, the dichotomization of the health traits helped us to relax the two central assumptions for the use of vignettes to assess health reporting behaviour, i.e. the shared understanding of the severity of health problems among respondents (‘vignette equivalence’) and the use of a consistent scale to evaluate the health of both the vignette characters and the respondents themselves (‘response consistency’).^11^ While the results of previous tests of these assumptions for SHARE vignettes were mixed,^31–33^ we found that our dichotomization approach satisfies the vignette equivalence assumption for almost all health traits and countries (results not shown but are available on request). Likewise, we do not expect any major problems with respect to response consistency, although it is not possible to validate this assumption on all health traits with the SHARE data.

It is important to emphasize that, due to these limitations and the compromises we had to make in the analysis, this study provides neither a correction nor an improvement of existing HLY estimates. This study must be considered as an attempt to provide only an approximate adjustment for DIF in estimating HLY. The primary research question we asked in this study was whether there are differences in health reporting behaviours across European populations, and if so, whether they can be a potential source of bias in the rankings of HLY. As seen in the consistency of the distribution of adjustment factors within the eight countries, the anchoring vignettes suggest that differences in health reporting styles indeed exist in the European context. Furthermore, our estimates of DIF-adjusted HLY indicate that DIF is likely to affect the differences in HLY between populations and country rankings of HLY, posing a considerable challenge to the comparability of HLY estimates across European countries.

Despite these limitations, this study represents an essential first step toward more accurately comparing population health in Europe. The analytical technique we employed is new and requires further improvements, but the presented findings underscore the importance of considering the role of DIF in intergroup comparisons of HLY. Fully understanding population health status requires research that addresses differences in health reporting behaviours among populations as well as population subgroups. Given that HE, as a summary indicator for population health, is quite important for health policies and public health measures, a failure to account for the role of DIF might lead to misleading conclusions.

## Supplementary Material

ckad142_Supplementary_Data

## Data Availability

Life tables from the Human Mortality Database are freely available at www.mortality.org. Limitations apply to the availability of data from SHARE and from EU-SILC. Microdata access can be requested at https://www.share-eric.eu/data/become-a-user and EU statistics on income and living conditions—Microdata—Eurostat (europa.eu), respectively.

## References

[1] Jagger C , Crimmins EM, Saito Y, et al International Handbook of Health Expectancies. Cham: Springer, 2020.

[2] Jagger C , McKee M, Christensen K, et al Mind the gap—reaching the European target of a 2-year increase in healthy life years in the next decade. Eur J Public Health 2013;23:829–33. 23487547 10.1093/eurpub/ckt030 PMC3784798

[3] Lagiewka K. European innovation partnership on active and healthy ageing: triggers of setting the headline target of 2 additional healthy life years at birth at EU average by 2020. Arch Public Health 2012;70:23. 23088612 10.1186/0778-7367-70-23 PMC3492155

[4] Jagger C , Gillies C, Moscone F, et al; EHLEIS Team. Inequalities in healthy life years in the 25 countries of the European Union in 2005: a cross-national meta-regression analysis. Lancet 2008;372:2124–31. 19010526 10.1016/S0140-6736(08)61594-9

[5] Jagger C. EU and UK targets for healthy life expectancy—are they achievable? Populationyearbook 2021;19:15–21.

[6] Fouweather T , Gillies C, Wohland P, et al; EHLEIS Team. Comparison of socio-economic indicators explaining inequalities in Healthy Life Years at age 50 in Europe: 2005 and 2010. Eur J Public Health 2015;25:978–83. 25876883 10.1093/eurpub/ckv070

[7] Janssen F , Mackenbach JP, Kunst AE; NEDCOM. Trends in old-age mortality in seven European countries, 1950–1999. J Clin Epidemiol 2004;57:203–16. 15125631 10.1016/j.jclinepi.2003.07.005

[8] Sauerberg M. The impact of population’s educational composition on Health Life Years: an empirical illustration of 16 European countries. SSM Popul Health 2021;15:100857. 34258376 10.1016/j.ssmph.2021.100857 PMC8255240

[9] Berger N , Van der Heyden J, Van Oyen H. The global activity limitation indicator and self-rated health: two complementary predictors of mortality. Arch Public Health 2015;73:25. 25964852 10.1186/s13690-015-0073-0 PMC4426645

[10] Van Oyen H , Bogaert P, Yokota RTC, Berger N. Measuring disability: a systematic review of the validity and reliability of the Global Activity Limitations Indicator (GALI). Arch Public Health 2018;76:25. 29881544 10.1186/s13690-018-0270-8 PMC5985596

[11] King G , Murray CJL, Salomon JA, Tandon A. Enhancing the validity and cross-cultural comparability of measurement in survey research. Am Polit Sci Rev 2004;98:191–207.

[12] Coenen J , Golsteyn BHH, Stolp T, Tempelaar D. Personality traits and academic performance: correcting self-assessed traits with vignettes. PLoS One 2021;16:e0248629. 33765063 10.1371/journal.pone.0248629 PMC7993818

[13] Angelini V , Cavapozzi D, Corazzini L, Paccagnella O. Age, health and life satisfaction among older Europeans. Soc Indic Res 2012;105:293–308. 22207782 10.1007/s11205-011-9882-x PMC3228960

[14] Kristensen N , Johansson E. New evidence on cross-country differences in job satisfaction using anchoring vignettes. Labour Econ 2008;15:96–117.

[15] Ravallion M , Himelein K, Beegle K. Can subjective questions on economic welfare be trusted? Evidence for three developing countries. World Bank Policy Res Work Pap 6726. The World Bank 2013.

[16] Kang E , Grol-Prokopczyk H. Comparing South Korean and US self-rated health using anchoring vignettes. Qual Life Res 2020;29:3213–22. 32770433 10.1007/s11136-020-02599-y

[17] Kapteyn A , Smith JP, van Soest AHO. Vignettes and self-reports of work disability in the United States and the Netherlands. Am Econ Rev 2007;97:461–73.

[18] Dowd JB , Todd M. Does self-reported health bias the measurement of health inequalities in U.S. adults? Evidence using anchoring vignettes from the Health and Retirement Study. J Gerontol B Psychol Sci Soc Sci 2011;66:478–89. 21666144 10.1093/geronb/gbr050

[19] Grol-Prokopczyk H , Freese J, Hauser RM. Using anchoring vignettes to assess group differences in general self-rated health. J Health Soc Behav 2011;52:246–61. 21673148 10.1177/0022146510396713 PMC3117438

[20] Oksuzyan A , Dańko MJ, Caputo J, et al Is the story about sensitive women and stoical men true? Gender differences in health after adjustment for reporting behavior. Soc Sci Med 2019;228:41–50. 30875543 10.1016/j.socscimed.2019.03.002

[21] Börsch-Supan A. Survey of Health, Ageing and Retirement in Europe (SHARE) Wave 1. Release version: 8.0.0. SHARE-ERIC, 2022. Data set. https://share-eric.eu/data/ (11 March 2023, date last accessed).

[22] Börsch-Supan A , Brandt M, Hunkler C, et al SHARE Central Coordination Team. Data resource profile: the Survey of Health, Ageing and Retirement in Europe (SHARE). Int J Epidemiol 2013;42:992–1001. 23778574 10.1093/ije/dyt088 PMC3780997

[23] Bergmann M , Kneip T, De Luca G, Scherpenzeel A. Survey participation in the Survey of Health, Ageing and Retirement in Europe (SHARE), Wave 1-7. Based on Release 7.0.0. SHARE Working Paper Series 41-2019. Munich: SHARE-ERIC, 2019.

[24] Sullivan DF. A single index of mortality and morbidity. HSMHA Health Rep 1971;86:347–54. 5554262 PMC1937122

[25] Wirth H , Pforr K. The European Union statistics on income and living conditions after 15 years. Eur Sociol Rev 2022;38:832–48.

[26] Human Mortality Database. Max Planck Institute for Demographic Research, University of California, Berkeley, and French Institute for Demographic Studies. Available at: https://www.mortality.org/ (20 February 2023, date last accessed).

[27] Idler E , Leventhal H, McLaughlin J, Leventhal E. In sickness but not in health: self-ratings, identity, and mortality. J Health Soc Behav 2004;45:336–56. 15595511 10.1177/002214650404500307

[28] Jylhä M. Self-rated health and subjective survival probabilities as predictors of mortality. In: Rogers RG, Crimmins EM, editors. International Handbook of Adult Mortality. Dordrecht: Springer, 2011: 329–44.

[29] Nocini R , Henry BM, Lippi G, Mattiuzzi C. Estimating the worldwide burden of health loss due to hearing loss. Eur J Public Health 2023;33:146–8. 36377968 10.1093/eurpub/ckac171 PMC9897997

[30] Elliott AF , McGwin G Jr, Kline LB, Owsley C. Vision impairment among older adults residing in subsidized housing communities. Gerontologist 2015;55 (Suppl 1):S108–17. 26055771 10.1093/geront/gnv028 PMC4566911

[31] Voňková H , Hullegie P. Is the anchoring vignette method sensitive to the domain and choice of the vignette? J R Stat Soc. Ser A Stat Soc 2011;174:597–620.

[32] d'Uva TB , O'Donnell O, van Doorslaer E. Differential health reporting by education level and its impact on the measurement of health inequalities among older Europeans. Int J Epidemiol 2008;37:1375–83. 18676985 10.1093/ije/dyn146 PMC2734070

[33] Rice N , Robone S, Smith P. Analysis of the validity of the vignette approach to correct for heterogeneity in reporting health system responsiveness. Eur J Health Econ 2011;12:141–62. 20349262 10.1007/s10198-010-0235-5

