# Supplementary material for: The impact of interpersonal reporting heterogeneity on cross-country differences in Healthy Life Years in Europe
Source: Eur J Public Health. 2023 Aug 22;33(6):1060–4. doi: 10.1093/eurpub/ckad142 (PMC10710331; doi:10.1093/eurpub/ckad142)
Supplement: ckad142_Supplementary_Data [file ckad142_supplementary_data.zip › ckad142_Supplementary_Data/ejph-2023-02-om-0092-File004.pdf]

Supplementary Material A

The anchoring vignettes module in SHARE is composed of two main parts. In questions V1-V7 respondents report their own health, answering questions about seven different health traits (“bodily aches or pains”, “difficulty with sleeping”, “problem with moving around”, “difficulty with concentrating and remembering”, “problem because of shortness of breath”, “problem with feeling sad, low, or depressed”, “limitations with the kind or amount of work one could do”). The response categories include “none,” “mild,” “moderate,” “severe,” and “extreme.” In the second part, respondents assess the health status of other fictive individuals over the same seven health traits, assuming the character described has the same age and background as their own. The vignettes in each domain were designed to depict varying degrees of health impairments, ranging from mild to severe conditions. SHARE includes in total 27 vignettes (questions V8-V34), three vignettes for each of the first six health traits and nine vignettes for the seventh health trait “limitations with the kind or amount of work one could do” (three each for limitations due to pain, depression, and stress and cardiovascular diseases). Respondents were asked to evaluate the severity of the described health problems with the same response categories as for the assessment of their own health.

Below we describe the vignettes related to the health trait based on bodily aches or pain as an example of the content of anchoring vignettes. A full list of SHARE vignettes can be found in https://www.share-eric.eu/data/data-documentation/questionnaires/wave-1

From the Self-Administered Questionnaire (SHARE 2004, type A Vignettes[1])

We would now like to ask you questions about your own health.

(Please choose one of the five answers for every question.)

1. Overall in the last 30 days, how much of bodily aches or pains did you have?

None	Mild	Moderate	Severe	Extreme
□1	□2	□3	□4	□5

We will now give you some examples of persons with serious and less serious health problems. We would like to know how you evaluate the health of these persons. Please assume that the persons have the same age and background that you have.

(Please choose one of the five answers for every question).

– Paul has a headache once a month that is relieved after taking a pill. During the headache he can carry on with his day-to-day affairs.

Overall in the last 30 days, how much of bodily aches or pains did Paul have?

None	Mild	Moderate	Severe	Extreme
□1	□2	□3	□4	□5

– Henri has pain that radiates down his right arm and wrist during his day at work. This is slightly relieved in the evenings when he is no longer working on his computer.

Overall in the last 30 days, how much of bodily aches or pains did Henri have?

None	Mild	Moderate	Severe	Extreme
□1	□2	□3	□4	□5

[1] Respondents of the vignettes questionnaire were randomized in two groups, A and B (see https://www.share-eric.eu/fileadmin/user_upload/Questionnaires/Q-Wave_1/w1_en_vignettes_A.pdf and https://www.share-eric.eu/fileadmin/user_upload/Questionnaires/Q-Wave_1/w1_en_vignettes_B.pdf for the English version). The two groups differ only in the order of the vignettes and the gender of the character.

– Charles has pain in his knees, elbows, wrists and fingers, and the pain is present almost all the time. Although medication helps, he feels uncomfortable when moving around, holding and lifting things.

Overall in the last 30 days, how much of bodily aches or pains did Charles have?

None	Mild	Moderate	Severe	Extreme
☐1	☐2	☐3	☐4	☐5
