# Supplementary material for: The impact of interpersonal reporting heterogeneity on cross-country differences in Healthy Life Years in Europe
Source: Eur J Public Health. 2023 Aug 22;33(6):1060–4. doi: 10.1093/eurpub/ckad142 (PMC10710331; doi:10.1093/eurpub/ckad142)
Supplement: ckad142_Supplementary_Data [file ckad142_supplementary_data.zip › ckad142_Supplementary_Data/ejph-2023-02-om-0092-File005.pdf]

Supplementary Material B

We followed a four-step procedure to obtain the adjustment factors w_{ijk}. This appendix provides a detailed description of the steps involved in computing the adjustment factors for German men aged 50-59, using the GALI indicator. To obtain the complete set of adjustment factors w_{ijk}, this procedure was replicated for all available countries, age groups (50-59, 60-74, 75 and above), and both genders. In step 1, we identified the health traits (V1-V7) associated with GALI for men aged 50-59. Then, we selected the vignettes related to those health traits (subset Q) from all available 27 vignettes (V8-V34) which were used to synthesize the difference in health reporting among countries (step 2 and 3), and lastly used to compute the adjusted HLY based on the adjusted GALI in step 4.

STEP 1: Identification of the health traits that are associated to the GALI

We run a least absolute shrinkage and selection operator (lasso) logistic regression on the GALI (dichotomized into “not limited at all”=healthy and “limited/severely limited”=unhealthy), using the health traits V1-V7 (dichotomized into “none/mild”=healthy and “moderate/severe/extreme”=unhealthy) as covariates and controlling by country of residence of respondents (Table B1). Male respondents who answer the questions V1-V7 and are aged 50-59 are included, irrespective of country of residence (Belgium, France, Germany, Greece, Italy, the Netherlands, Spain, Sweden). The lasso logistic regression estimates model coefficients and determines which covariates to keep in a model to obtain the best prediction of the variable of interest[1].

[1] Hastie, Trevor, Robert Tibshirani, and Martin Wainwright. 2015. Statistical Learning with Sparsity: The Lasso and Generalizations. New York: Chapman and Hall/CRC (https://doi.org/10.1201/b18401).

The results show that for men aged 50-59, pain, sleeping issues, depression, and limitations (in the amount of work they can do) were the health traits selected by the lasso procedure. This information is used in the next step.

Table B1 Results of the lasso logistic regression on GALI, men, 50-59

Vignette health trait	Selected
V1 - Bodily aches or pains	X
V2 - Difficulty with sleeping	X
V3 - Problem with moving around	
V4 - Difficulty with concentrating and remembering	
V5 - Problem because shortness of breath	
V6 - Problem with feeling sad, low, or depressed	X
V7 - Limitations with the kind or amount of work one could do	X

Data: SHARE 2004; authors' own estimations

Note: controlled by country; selection based on the Bayesian Information Criterion (BIC).

STEP 2: Selection of vignettes

We selected only those vignettes that refer to the health traits that were identified in step 1 to be associated with GALI among men aged 50-59 from the total sample of all eight countries, i.e., the vignettes related to pain (V8, V10, and V12), sleeping problems (V9, V11, and V13), depression (V21, V23, and V25), and limitations in the work one can do (V26-V34). This subset of vignettes (indicated as Q in the methods section) was used to calculate the percentage of respondents of all ages together who assessed the described health problem as "moderate/severe/extreme", separately for each country (denominator of the calculation formula,

see methods section). The corresponding value for the total of all eight countries was set as the standard value (enumerator of the calculation formula), to which we relate the values of each country.

There is a significant variation in the level of severity attributed to the same health-related characteristics across different countries. The proportion of men who assessed the health problem of the selected vignette characters as moderate, severe, or extreme is 79.2% in Germany, 87.9% in Sweden, 75.2% in the Netherlands, 82.6% in Spain, 68.4% in Italy, 77.5% in France, 77.7% in Greece, and 76.1% in Belgium. The corresponding value for all countries taken together is 77.8%. Consequently, respondents from Sweden and Spain assess certain traits as more problematic than respondents from the Netherlands and Italy. Germany, France, Greece, and Belgium have similar assessments as the average of all countries.

STEP 3 – Computation of adjustment factors

In the third step of the analysis, the adjustment factors are calculated by dividing the value of 77.8 for all countries by the corresponding value of the respective country. In the case of men aged 50-59 from Germany, this results in a value of 0.982 (=77.8/79.2). This value is close to one, indicating that the pattern of answers is similar to the average value of the pooled countries sample. Values exceeding 1.0 (e.g., Italy with 77.8/68.4=1.14) suggest that the specific country sample is more optimistic in assessing a given health condition than the total sample of all countries. Conversely, values below 1.0 (e.g., Sweden with 77.8/87.9=0.88) indicate that the respondents from the specific country are more pessimistic in assessing the same health condition than the total sample.

STEP 4 – Adjustment of the GALI, by age, gender, country

The variations in health vignette ratings across countries reflect the heterogeneity in health reporting. Therefore, the adjustment factors derived in step 3 can be used to approximately adjust for this heterogeneity and to standardize the country-specific GALI prevalence values to the level of the total sample's reporting behavior by multiplying the observed GALI values with adjustment factors. This procedure increases or decreases the proportion of people with limitations if the value of the adjustment factor is higher or lower than 1.0, respectively. Table B2 reports the results for the example of men from Germany aged 50-59.

Table B2 Computation of adjusted GALI, Germany, men, 50-59

Germany, men (age)	Unadjusted GALI (% limited)	$w_{men,\ 50\text{-}59,\ Germany}$	Adjusted GALI (% limited)
	A	B	A*B
50-54	0.370	0.982	0.363
55-59	0.453	0.982	0.445

Source: Authors' own calculations based on the data from the SHARE 2004, EU-SILC 2005, and HMD 2005.

Repeating step 1 for all age groups and both genders and steps 2-4 for all age groups, both genders and countries provide the full set of adjustments factors.
