# Supplementary material for: The impact of interpersonal reporting heterogeneity on cross-country differences in Healthy Life Years in Europe
Source: Eur J Public Health. 2023 Aug 22;33(6):1060–4. doi: 10.1093/eurpub/ckad142 (PMC10710331; doi:10.1093/eurpub/ckad142)
Supplement: ckad142_Supplementary_Data [file ckad142_supplementary_data.zip › ckad142_Supplementary_Data/ejph-2023-02-om-0092-File006.pdf]

Supplementary Material C

Table C1 Vignette health traits associated with GALI limitations; results derived from lasso logistic regression modelling of health traits on GALI; by age group among women (W) and men (M)

Vignette health trait	Age group		
	50-59	60-74	75+
V1 - Bodily aches or pains	W, M	W, M	W, M
V2 - Difficulty with sleeping	W, M		
V3 - Problem with moving around	W	W, M	W, M
V4 - Difficulty with concentrating and remembering			
V5 - Problem because shortness of breath	W	W, M	
V6 - Problem with feeling sad, low, or depressed	M	W	
V7 - Limitations with the kind or amount of work one could do	W, M	W, M	W, M

Data: SHARE 2004; authors' own estimations

Notes: GALI limitations were defined by the answer categories "strongly limited" and "limited, but not strong", health problems in the vignette health traits were defined by the answer categories "moderate", "severe" and "extreme"; weighted values; all models controlled by country.
